# Hypertension control among euvolemic hypertensive hemodialysis patients in Malaysia: a prospective follow-up study

**DOI:** 10.1186/s40545-019-0169-y

**Published:** 2019-05-14

**Authors:** Amjad Khan, Amer Hayat Khan, Azreen Syazril Adnan, Syed Azhar Syed Sulaiman, Saima Mushtaq, Nafees Ahmad, Irfanullah Khan

**Affiliations:** 1 Discipline of Clinical Pharmacy, School of Pharmaceutical Sciences, Universiti Sains Malaysia, 11800 Penang, Malaysia; 2 Chronic Kidney Disease Resource Centre, School of Medical Sciences, Health Campus, Universiti Sains Malaysia, 16150 Kubang Kerian, Kelantan Malaysia; 3 Department of Pharmacy, Quaid-i-Azam University, Islamabad, 45320 Pakistan; 4 Health Care Biotechnology Department, Atta ur Rahman School of Applied Biosciences, National University of Sciences & Technology, Islamabad, 44000 Pakistan; 5 Faculty of Pharmacy and Health Sciences, University of Balochistan, Quetta, 87300 Pakistan

**Keywords:** Calcium channel blockers, Euvolemic, Hemodialysis, Hypertension

## Abstract

**Objectives:**

Existing literature does not provide enough information on evaluation of factors associated with pre-dialysis controlled hypertension among euvolemic hemodialysis (HD) patients. We conducted a study to evaluate the rate and factors influencing pre-dialysis controlled hypertension among euvolemic HD patients.

**Design:**

A multicenter prospective follow-up study.

**Setting:**

Tertiary care teaching hospital and its associated private dialysis centers.

**Participants:**

This study included 145 euvolemic eligible hypertensive patients. Various sociodemographic, clinical factors and drugs were investigated and analyzed by using appropriate statistical methods to determine the factors influencing hypertension control among the study participants.

**Results:**

On baseline visit, the mean pre-dialysis systolic and diastolic BP (mmHg) of study participants was 161.2 ± 24. and 79.21 ± 11.8 retrospectively, and 30 (20.6%) patients were on pre-dialysis goal BP. At the end of the 6-months follow-up, the mean pre-dialysis systolic BP and diastolic BP (mmHg) of the patients was 154.6 ± 18.3 and 79.2 ± 11.8 respectively, and 42 (28.9%) were on pre-dialysis goal BP. In multivariate analysis, the use of calcium channel blockers (CCBs) was the only variable which had statistically significant association with pre-dialysis controlled hypertension at baseline (OR = 7.530, *p*-value = 0.001) and final (OR = 8.988, *p*-value < 0.001) visits.

**Conclusions:**

In present study**,** the positive association observed between CCBs and controlled hypertension suggests that CCBs are effective antihypertensive drugs in the management of hypertension among euvolemic HD patients.

**Strengths and limitations of this study:**

- This study involved a group of patients from tertiary-level teaching hospital and its associated private dialysis centers of Malaysia.
- To the best of the authors’ knowledge, this is the first study to assess the factors influencing pre-dialysis controlled hypertension in a cohort of 145 euvolemic HD patients in a Malaysian setting.
- For determining the factors influencing hypertenion control multivariate analysis was conducted.
- Being a prospective follow-up study, the findings of the present study need to be interpreted with caution since it is limited to only 6 months follow up.
- Nevertheless, a multicenter study with a large sample size and longer follow up time is needed to confirm the findings of the current study.

## Background

Hypertension is common and often poorly controlled among hemodialysis (HD) patients. In fact, volume overload is considered as an important cause of hypertension where patients may remain hypertensive even after thrice weekly HD sessions. In such patients, non-volume mechanisms such as activation of the renin angiotensin system and/or sympatho-adrenal activities, are important contributors to hypertension [1–3]. Due to their safety, tolerability and good therapeutic efficacies, renin angiotensin aldosterone system (RAAS) inhibitors are also considered as the first line agents in the treatment of hypertension among HD patients [4]. The national kidney foundation disease outcomes quality initiative (KDOQI) guidelines also recommend the use of RAAS inhibitors among dialysis patients having diabetic and heart failures [5].

A literature suggests that systolic BP is associated with cardiovascular adverse events [6]. Studies by Moist et al. and Efrati et al. concluded that the use of angiotensin converting enzyme (ACE) inhibitors is associated with improved survival [7, 8]. In fact, blood pressure (BP) control and cardiovascular outcomes can be improved by combining ACE inhibitors and angiotensin receptor blockers (ARBs) therapies [9]. Calcium channel blockers (CCB)s and other vasodilators are also considered to be effective in managing BP where CCBs are often widely applied in patients with volume overload and can very useful for lowering the BP among HD patients [10]. A recent randomized controlled trial reported that amlodipine can lower systolic BP ∼ 10 mmHg as compared with placebo (7% vs. 13%, respectively) without introducing an intradialytic hypotension [11]. Nevertheless, there is limited literature available on the role of CCBs regarding the management of hypertension among HD patients.

Among the general population, studies investigating CCBs use indicated mixed findings regarding their effects on patient’s outcome [12–18]. For example, the use of short acting dihydropyridines leads to a higher risk of developing myocardial infarction while the longer acting CCBs pose some mortality risks as also seen with the use of other antihypertensive medications [10]. Generally, CCBs are commonly prescribed to patients with end stage renal disease (ESRD), mainly for BP control though it may have different effects in ESRD patients [10]. CCBs inhibit vasoconstriction as well as both the hypertrophic and hyperplastic effects of angiotensin II and other mitogens on the mesangial and vascular smooth muscle cells by blocking calcium-dependent mechanisms [19–21]. The USA national clinical practice guideline (2005), recommended a pre-dialysis BP of less than 140/90 mmHg and post-dialysis BP of less than 130/80 mmHg [22]. However, achieving these standards in clinical practice remains a challenge. In this study, an observational analysis to evaluate the factors influencing pre-dialysis controlled hypertension among euvolemic HD patients is conducted.

## Materials and methods

### Study location and participants

This was a multicenter, prospective follow-up study conducted among HD patients at Hospital Universiti Sains Malaysia (HUSM), which is a tertiary care hospital and its associated dialysis centers in Kelantan, Malaysia. All confirmed hypertensive HD patients between 1st April 2017 to 31st December 2017 who received anti-hypertensives and have to undergo dialysis three times a week were consecutively enrolled in the study.

### Operational definitions

#### Hypertension

According to KDOQI guidelines, pre-dialysis and postdialysis BP goals should be < 140/90 mmHg and < 130/80 mmHg, respectively.

### Controlled hypertension

Patients with a mean systolic/diastolic BP of < 130/80 mmHg were considered as having controlled hypertension.

### Hypervolemia, Euvolemia and hypovolemia

A multi-frequency (5–1000 kHz) portable bioimpedance spectroscopy device (Body Composition Monitor, BCM, Fresenius Medical Care, Germany) was used to assess fluid status. The BCM-calculated overhydration (OH) value was used as a fluid overload indicator. Accordingly, OH > 1.1 L was categorized as fluid overload or hypervolemia. An OH value lower than the 10th percentile (− 1.1 L) was defined as hypovolemia. An OH value of ±1.0 L was defined as euvolemia, i.e., normal hydration status [23–25].

**Fig. 1:**
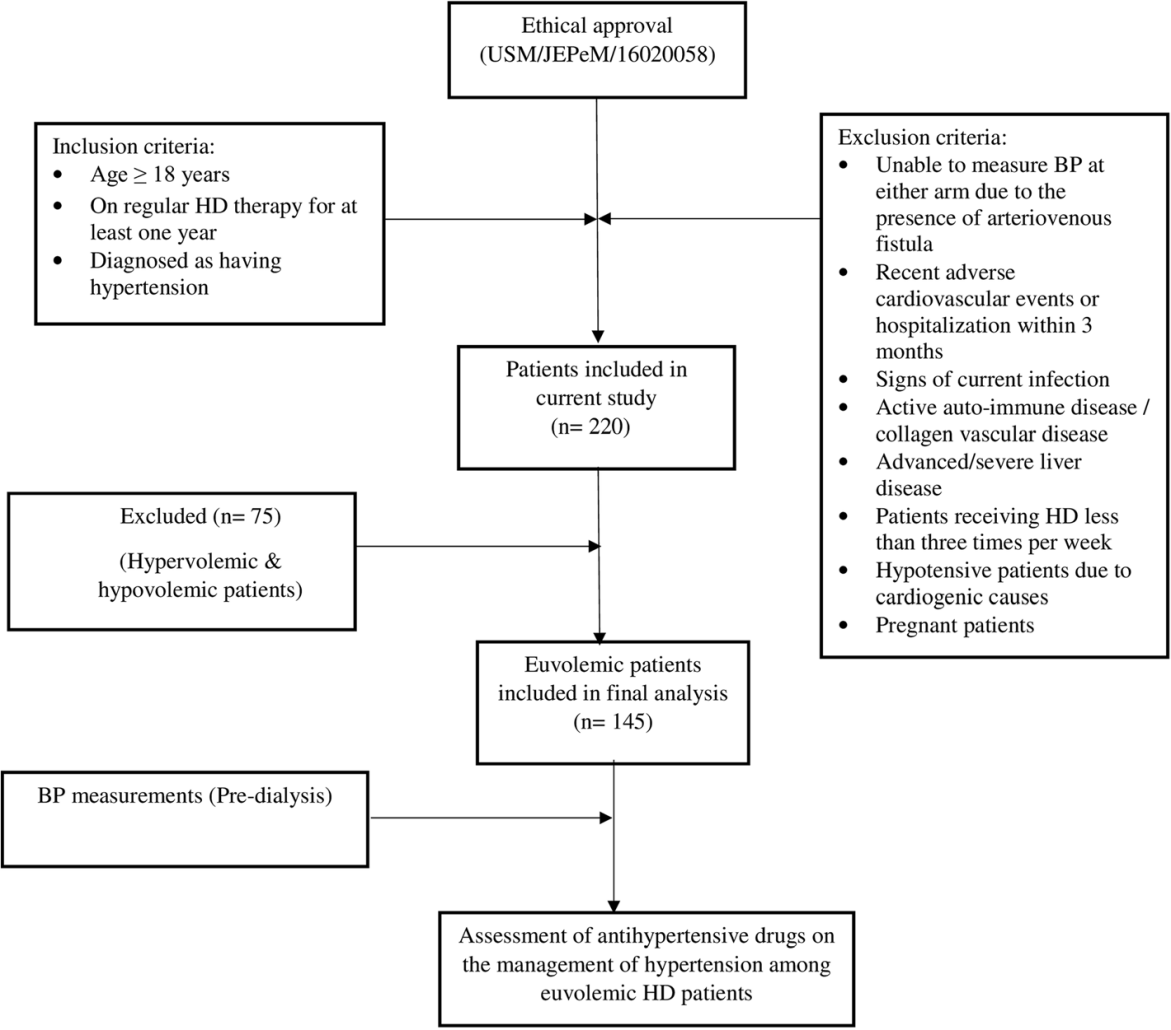
Study Design

Patients with pre-dialytic hypotension (having a systolic BP less than 110 mmHg) or high BP > 200/100 mmHg were excluded from the study. A total of 220 met the eligibility criteria and were included in the study **(**Fig. 1). From this number, 75 hyper and hypovolemic patients were excluded. Finally, the pre-dialysis BP measurements and the effect of antihypertensive drugs on BP on 145 euvolemic patients were assessed. The study procedures were in accordance with the clinical practice guidelines for HD from National Kidney Foundation Kidney Disease Outcome Quality Initiative (NKF KDOQI) [26]. Diagnosis of cardiovascular disease and other comorbidities were based on documentation from patient’s medical record. Patients with ischemic heart disease, heart failure and left ventricular hypertrophy were considered to have cardiovascular disease. We used the criteria based on advisory committee suggestions, extensive literature review, hypothetical possible association and nephrologist’s suggestions i.e. If three consecutive BCM readings confirms the euvolemic state, then those patients are considered as euvolemic HD patients and we further proceeded them for hypertension evaluation.

### Data collection

Both socio-demographic and clinical data were collected from the regularly updated Advanced Dialysis Nephrology Application Network (ADNAN) at the study sites (URL: http://www.microsemi.com.my/product/advanced-dialysis-nephrologist-application-network-adnan-system) using a standardized data collection form. Height, weight and BP were measured during the physical examination. Only a single calibrated manual sphygmomanometer was used to measure BP in all of the patients. A multi-frequency (5–1000 kHz) portable bioimpedance spectroscopy device (Body Composition Monitor, BCM, Fresenius Medical Care, Germany) was used to assess fluid status.

On the dialysis day, pre-dialysis BP was taken as a mean of three consecutive measurements with 5-min intervals. BP was recorded by a senior member of the nursing staff dedicated to the study. As per KDOQI guidelines, BP goals were defined as < 140/90 and < 130/80 mmHg for pre-and post-dialysis respectively. Patients with a mean systolic/diastolic BP of > 140/90 mmHg were considered as having an uncontrolled hypertension. During the 6 months’ follow-up, the mean pre-dialysis BP readings at baseline, 1, 2, 3, 4, 5 and 6 months were recorded and the effects of antihypertensive drugs on pre-dialysis BP control were assessed.

### Statistical analysis

Statistical Package for Social Sciences (SPSS version 21, Chicago, USA) was used for data analysis. Means and standard deviations were calculated for continuous variables, whereas categorical variable are presented as frequencies and percentages. Chi-squared test was used to observe significance between categorical variables. Multivariate logistic regression analysis with the Wald statistical criteria was used to obtain a final model. A *p*-value of < 0.05 was considered statistically significant. Relevant variables with a *p*-value < 0.25 in the univariate analysis were included in the multivariate analysis. We confirmed the correlations among variables entered in the multivariate analysis. The results of multivariate analysis were presented as beta, standard error, *p*-value, adjusted odds ratio and 95% confidence interval. The fit of the model was assessed by Hosmer Lemeshow and overall classification percentage.

## Results

The mean age of the study participants (*n* = 145) was 58.68 (± 9.86) years. The majority were females (51.7%), 41–60 years old (57.2%), of a normal body mass index (BMI) (62.1%) and on dialysis for more than 5 years (31%). Since the study was conducted in the Malaysian state of Kelantan, most patients were of Malay ethnicity (96.6%) **(**Table 1).

**Table 1:** Baseline demographics and characteristics of euvolemic hemodialysis patients (*n* = 145)

Variables	No. (%)
Gender	
Female	75 (51.7)
Male	70 (48.3)
Age mean (±SD)	58.68 (± 9.857)
Age group (years)	
< 40	7 (4.8)
41–60	83 (57.2)
> 60	55 (37.9)
BMI mean (±SD)	23.908 (± 4.3505)
BMI classification	
Underweight	6 (4.1)
Normal	90 (62.1)
Overweight	42 (29)
Obese	7 (4.8)
Education	
Uneducated	40 (27.6)
Educated	105 (72.4)
Marital status	
Single	6 (4.1)
Married	139 (95.9)
Ethnicity	
Malay	140 (96.6)
Others	5 (3.4)
Smoking status	
Current Smoker	44 (30.3)
Non-Smoker	101 (69.7)
Alcohol	
Current drinker	6 (4.1)
Non-drinker	139 (95.9)
Drug addiction	
Current Drug Addiction	16 (11)
No Drug Addiction	129 (89)
Employment	
Unemployed	77 (53.1)
Employed	68 (46.9)
Years of dialysis	
1 year	43 (29.7)
2–4 years	57 (39.3)
> 5 years	45 (31)
Hemodialysis centers	
Private	84 (57.9)
NGO	24 (16.6)
Governmental	37 (25.5)
Vascular access	
Fistula	135 (93.1)
Others	10 (6.9)
Diabetes mellitus	
No	48 (33.1)
Yes	97 (66.9)
Cardiovascular diseases	
No	125 (86.2)
Yes	20 (13.8)
Cerebrovascular accident	
No	131 (90.3)
Yes	14 (9.7)
Hyperlipidemia	
No	125 (86.2)
Yes	20 (13.8)
Gouty arthritis	
No	127 (87.6)
Yes	18 (12.4)
Other comorbidities^a^	
No	104 (71.7)
Yes	41 (28.3)

*SD* Standard deviation, *BMI* Body Mass Index, *NGO* Non-governmental organization

^a^Other comorbidities: Blood clots, depression, asthma, osteoarthritis, pregnancy losses/birth defects and osteoporosis

The most common comorbidities were hypertension (*n* = 118, 81.3%) and diabetes (*n* = 97, 66.9%). Calcium channel blockers (*n* = 43, 29.7%) was the most prescribed antihypertensive followed by beta antagonist (*n* = 42, 29%). Table 2 gives an account for euvolemic hemodialysis patients comorbid conditions and antihypertensive medication.

**Table 2:** Euvolemic hemodialysis patient’s antihypertensive medication and comorbidities (*n* = 145)

Patient variables	No. (%)
Antihypertensive medication	
ACE-I	12 (8.3)
ARBs	36 (24.8)
CCBs	43 (29.7)
Alpha antagonist	6 (4.1)
Beta antagonist	42 (29)
Diuretics	41 (28.3)
Antihypertensive combination therapy	15 (10.3)
Co-morbid conditions	
Hypertension	118 (81.3)
Diabetes mellitus	97 (66.9)
Cardiovascular diseases	20 (13.8)
Cerebrovascular accident	14 (9.7)
Hyperlipidemia	20 (13.8)
Gouty arthritis	18 (12.4)
Other comorbidities^a^	41 (28.3)

*ACE-I* Angiotensin converting enzyme inhibitors, *ARBs* Angiotensin receptor blockers, *CCBs* Calcium channel blockers, ^a^Other comorbidities: Blood clots, depression, asthma, osteoarthritis, pregnancy losses/birth defects and osteoporosis

### Overall blood pressure changes

At the baseline visit, the mean pre-dialysis systolic BP was 161.2 ± 24.9 mmHg while pre-dialysis diastolic BP was 79.21 ± 11.8 mmHg at baseline. At the end of the 6-months follow-up, the mean pre-dialysis systolic BP was 154.6 ± 18.3 mmHg giving a change in BP of − 6.6 mmHg. Similarly, pre-dialysis diastolic BP which was 79.21 ± 11.8 mmHg at baseline, dropped to 75.0 mmHg ±7.2 mmHg at the end of study; a difference of − 4.2 mmHg. The mean pulse rate was 78 ± 13.9 beats per min at baseline which decreased to 74.5 ± 10.4. The mean baseline interdialytic weight gain was 1.8 ± 0.8 kg with only 1.5 ± 0.5 kg mean interdialytic weight gain at the end of study (Table 3).

**Table 3:** Blood Pressure readings during the course of study (n = 145)

Variables	Baseline Mean (±SD)	1st month Mean (±SD)	2nd month Mean (±SD)	3rd month Mean (±SD)	4th month Mean (±SD)	5th Month Mean (±SD)	6th Month Mean (±SD)
Pre-dialysis systolic BP	161.2 (±24.9)	159.2 (±23.3)	158.0 (±21.9)	157.1 (±21.0)	156.4 (±20.1)	155.7 (±19.5)	154.6 (±18.3)
Pre-dialysis diastolic BP	79.2 (±11.8)	78.3 (±10.7)	77.5 (±9.5)	77.2 (±9.1)	76.5 (±8.4)	75.9 (±7.8)	75.0 (±7.2)
Pre-dialysis pulse rate	78.0 (±13.9)	77.2 (±12.9)	76.6 (±12.0)	76.1 (±11.4)	75.7 (±11.2)	75.15 (±10.8)	74.5 (±10.4)
Interdialytic weight gain	1.8 (±0.8)	1.8 (±0.7)	1.6 (±0.6)	1.5 (±0.5)	1.6 (±0.6)	1.4 (±0.5)	1.3 (±0.4)

**Fig. 2:**
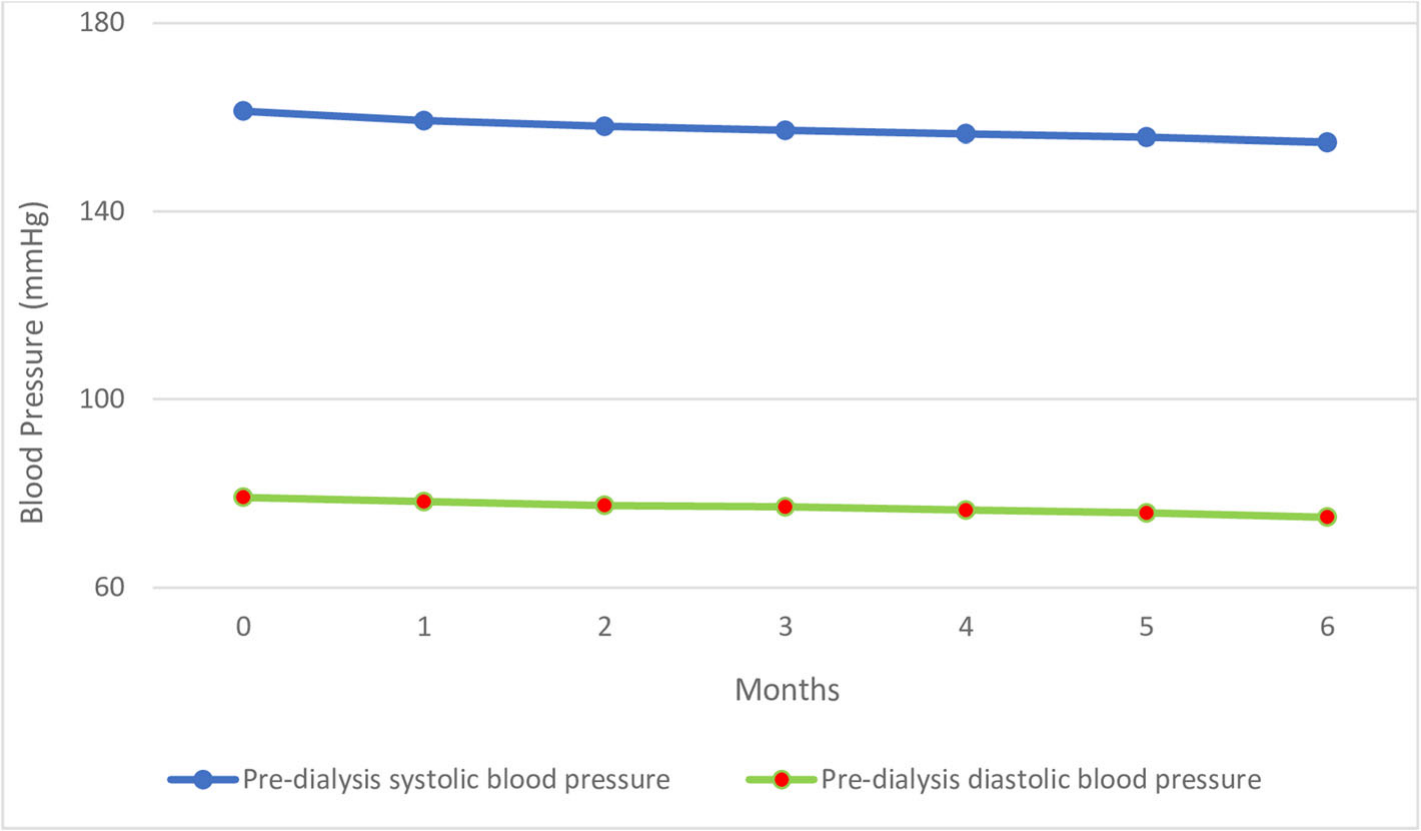
Graphical representation of pre-dialysis blood pressure variations during the six month's follow up

Pre-dialysis BP variations of study duration are presented in graphical form in Fig. 2. There is a linear decrease in both mean systolic and diastolic BP from baseline towards sixth month.

### Overall mean blood pressure readings of all visits of study participants

At the end of 6-month patient follow-up, the mean readings of all visits were calculated. The mean pre-dialysis systolic BP of all visits was 157.4 ± 2.2 mmHg and pre-dialysis diastolic BP was 77.0 ± 1.4 mmHg. Mean pulse rate was 76.1 ± 1.2 beats/min and mean interdialytic weight gain was reported as 1.5 ± 0.1 kg at the end of study. Table 4 provides the overall mean BP readings.

**Table 4:** Overall mean BP readings during the course of study (*n* = 145)

Variables	Mean (±SD)
Pre-dialysis systolic (mean of all BP readings)	157.4 (±2.2)
Pre-dialysis diastolic (mean of all BP readings)	77.0 (±1.4)
Pre-dialysis pulse rate (mean of all readings)	76.1 (±1.2)
Interdialytic weight gain (mean of all readings)	1.5 (±0.1)

### Univariate and multivariate analysis (baseline)

On baseline visit, a total of 30 (20.6%) patients were on pre-dialysis goal BP of <130/80 mmHg. Upon univariate binary logistic regression analysis, the associations observed between various independent variables and pre-dialysis controlled hypertension at baseline visit are given in **(**Table 5).

**Table 5:** Univariate and multivariate analysis of factors associated with pre-dialysis controlled hypertension at baseline (*n* = 145)

Variables	Patients with pre-dialysis controlled hypertension at baseline Number (%)		Univariate analysis OR (95% CI)	*p*-value	Multivariate analysis OR (95% CI)	*p*-value
	Yes	No				
Gender						
Female	18 (24)	57 (76)	Reference			
Male	12 (17.1)	58 (82.9)	0.655 (0.289-1.483)	0.310		
Age						
≤ 40	1 (14.3)	6 (85.7)	Reference			
41-60	15 (18.1)	68 (81.9)	1.324 (0.148-11.821)	0.802		
> 60	14 (25.5)	41 (74.5)	2.049 (0.227-18.531)	0.523		
BMI						
Underweight	3 (50)	3 (50)	Reference		Reference	
Normal	17 (18.9)	73 (81.1)	0.233 (0.043-1.256)	0.090	0.597 (0.080-4.470)	0.615
Overweight	7 (16.7)	35 (83.3)	0.200 (0.033-1.203)	0.079	0.378 (0.036-3.951)	0.417
Obese	3 (42.9)	4 (57.1)	0.750 (0.084-6.710)	0.797	1.737 (0.109-1.851)	0.268
Smoking status						
Current Smoker	8 (18.2)	36 (81.8)	Reference			
Non-Smoker	22 (21.8)	79 (78.2)	1.253 (0.509-3.083)	0.623		
Drug addiction						
Current drug addiction	5 (31.3)	11 (68.8)	Reference			
No drug addiction	25 (19.4)	104 (80.6)	0.529 (0.169-1.660)	0.275		
Hemodialysis centers						
Private	16 (19)	68 (81)	Reference		Reference	
NGO	3 (12.5)	21 (87.5)	0.607 (0.161-2.288)	0.461	0.448 (0.109-1.851)	0.268
Governmental	11 (29.7)	26 (70.3)	1.798 (0.738-4.382)	0.197	0.959 (0.299-3.077)	0.944
Vascular access						
Fistula	28 (20.7)	107 (79.3)	Reference			
Others	2 (20)	8 (80)	0.955 (0.192-4.753)	0.956		
Diabetes mellitus						
No	10 (20.8)	38 (79.2)	Reference			
Yes	20 (20.6)	77 (79.4)	0.987 (0.421-2.316)	0.976		
Cardiovascular diseases						
No	26 (20.8)	99 (79.2)	Reference			
Yes	4 (20)	16 (80)	0.952 (0.293-3.091)	0.935		
Cerebrovascular accident						
No	27 (20.6)	104 (79.4)	Reference			
Yes	3 (21.4)	11 (78.6)	1.051 (0.274-4.032)	0.943		
Hyperlipidemia						
No	30 (24)	95 (76)	Non-computable			
Yes	–	20 (100)		–		
Gouty arthritis						
No	29 (22.8)	98 (77.2)	Reference		Reference	
Yes	1 (5.6)	17 (94.4)	0.199 (0.25-1.558)	0.124	0.304 (0.033-2.770)	0.291
Others comorbidities^a^						
No	18 (17.3)	86 (82.7)	Reference		Reference	
Yes	12 (29.3)	29 (70.7)	1.977 (0.851-4.593)	0.113	2.307 (0.851-6.260)	0.101
ACE-I						
No	30 (22.6)	103 (77.4)	Non-Computable			
Yes	–	12 (100)		–		
ARB						
No	20 (18.3)	89 (81.7)	Reference		Reference	
Yes	10 (27.8)	26 (72.2)	1.712 (0.713-4.109)	0.229	3.436 (0.965-12.238)	0.061
CCB						
No	13 (12.7)	89 (87.3)	Reference		Reference	
Yes	17 (39.5)	26 (60.5)	4.476 (1.925-10.411)	0.001	7.530 (2.413-23.498)	0.001
Alpha antagonist						
No	27 (19.4)	112 (80.6)	Reference		Reference	
Yes	3 (50)	3 (50)	4.148 (0.793-21.698)	0.092	4.049 (0.510-32.120)	0.186
Beta antagonist						
No	19 (18.4)	84 (81.6)	Reference			
Yes	11 (26.2)	31 (73.8)	1.569 (0.671-3.667)	0.299		
Diuretics						
No	23 (22.1)	81 (77.9)	Reference			
Yes	7 (17.1)	34 (82.9)	0.725 (0.284-1.849)	0.501		
Other combination of antihypertensives						
No	27 (20.8)	103 (79.2)	Reference			
Yes	3 (20)	12 (80)	0.954 (0.251-3.621)	0.944		
Type of therapy						
Mono-therapy	15 (19)	64 (81)	Reference			
Multi-therapy	15 (22.7)	51 (77.3)	1.255 (0.561-2.806)	0.580		
Statins						
No	8 (20)	32 (80)	Reference			
Yes	22 (21)	83 (79)	1.060 (0.428-2.624)	0.899		
Phosphate binders						
No	5 (27.8)	13 (72.2)	Reference			
Yes	25 (19.7)	102 (80.3)	0.637 (0.208-1.954)	0.431		

Analysis: Univariate and Multivariate binary logistic regression analysis. All variables with *p*-value < 0.25 will be included in the multivariate analysis. *OR* Odds ratio, *CI* confidence interval, *BMI* Body mass index, *NGO* Non-governmental organization, ^a^Other comorbidities: Blood clots, depression, asthma, osteoarthritis, pregnancy losses/birth defects and osteoporosis. *ACE-I* Angiotensin converting enzyme inhibitors, *ARB* Angiotensin receptor blocker, *CCB* Calcium channel blocker

In the multivariate logistic regression analysis, the only variable which was statistically significant associated with pre-dialysis controlled hypertension was the use of CCBs (OR = 7.530, *p*-value = 0.001) **(**Table 5).

### Univariate and multivariate analysis (upon study completion)

Upon final visit, a total of 42 (28.9%) patients were on pre-dialysis goal BP of <130/80 mmHg. Upon univariate binary logistic regression analysis, the associations observed between various independent variables and pre-dialysis controlled hypertension at final visit are given in Table 6.

**Table 6:** Univariate and multivariate analysis of factors associated with pre-dialysis controlled hypertension upon study completion (*n* = 145)

Variables	Patients with pre-dialysis controlled hypertension on final visit Number (%)		Univariate analysis OR (95% CI)	*p*-value	Multivariate analysis OR (95% CI)	*p*-value
	Yes	No				
Gender						
Female	20 (26.7)	55 (73.3)	Reference			
Male	22 (31.4)	48 (68.6)	1.260 (0.614-2.586)	0.528		
Age						
≤ 40	3 (42.9)	4 (57.1)	Reference			
41-60	22 (26.5)	61 (73.5)	0.481 (0.100-2.321)	0.362		
> 60	17 (30.9)	38 (69.1)	0.596 (0.120-2.962)	0.527		
BMI						
Underweight	3 (50)	3 (50)	Reference		Reference	
Normal	22 (24.4)	68 (75.6)	0.324 (0.061-1.720)	0.186	0.809 (0.123-5.330)	0.826
Overweight	13 (31)	29 (69)	0.448 (0.080-2.526)	0.363	0.705 (0.084-5.927)	0.747
Obese	4 (57.1)	3 (42.9)	1.333 (0.149-11.929)	0.797	4.775 (0.352-64.836)	4.775
Smoking status						
Current Smoker	12 (27.3)	32 (72.7)	Reference			
Non-Smoker	30 (29.7)	71 (70.3)	1.127 (0.512-2.480)	0.767		
Drug addiction						
Current drug addiction	8 (50)	8 (50)	Reference		Reference	
No drug addiction	34 (26.4)	95 (73.6)	0.358 (0.125-1.028)	0.056	0.492 (0.129-1.870)	0.298
Hemodialysis centers						
Private	20 (23.8)	64 (76.2)	Reference		Reference	
NGO	7 (29.2)	17 (70.8)	1.318 (0.478-3.630)	0.594	1.130 (0.302-4.224)	0.856
Governmental	15 (40.5)	22 (59.5)	2.182 (0.955-4.985)	0.064	1.909 (0.601-6.062)	0.273
Vascular access						
Fistula	38 (28.1)	97 (71.9)	Reference			
Others	4 (40)	6 (60)	1.702 (0.455-6.368)	0.430		
Diabetes mellitus						
No	18 (37.5)	30 (62.5)	Reference		Reference	
Yes	24 (24.7)	73 (75.3)	0.548 (0.260-1.154)	0.113	0.415 (0.156-1.105)	0.078
Cardiovascular diseases						
No	26 (20.8)	99 (79.2)	Reference			
Yes	4 (20)	16 (80)	0.793 (0.269-2.340)	0.674		
Cerebrovascular accident						
No	37 (29.6)	88 (70.4)	Reference			
Yes	5 (25)	15 (75)	0.643 (0.170-2.434)	0.516		
Hyperlipidemia						
No	41 (32.8)	84 (67.2)	Reference			
Yes	7 (35.0)	13 (65)	0.830 (0.730-2.903)	0.830		
Gouty arthritis						
No	39 (30.7)	88 (69.3)	Reference		Reference	
Yes	3 (16.7)	15 (83.3)	0.451 (0.124-1.649)	0.229	1.312 (0.213-8.094))	0.770
Others comorbidities^a^						
No	26 (25)	78 (75)	Reference		Reference	
Yes	16 (39)	25 (61)	1.920 (0.890-4.141)	0.096	1.865 (0.690-5.041)	0.219
ACE-I						
No	39 (29.3)	94 (70.7)	Reference			
Yes	3 (25)	9 (75)	0.803 (0.206-3.127)	0.752		
ARB						
No	31 (28.4)	78 (71.6)	Reference			
Yes	11 (30.6)	25 (69.4)	1.107 (0.487-2.519)	0.808		
CCB						
No	17 (16.7)	85 (83.3)	Reference		Reference	
Yes	25 (58.1)	18 (41.9)	6.296 (2.843-13.943)	< 0.001	8.988 (3.140-25.728)	< 0.001
Alpha antagonist						
No	40 (28.8)	99 (71.2)	Reference			
Yes	2 (33.3)	4 (66.7)	1.237 (0.218-7.027)	0.810		
Beta antagonist						
No	28 (27.2)	75 (72.8)	Reference			
Yes	14 (33.3)	28 (66.7)	1.339 (0.617-2.906)	0.460		
Diuretics						
No	34 (32.7)	70 (67.3)	Reference		Reference	
Yes	8 (19.5)	33 (80.5)	0.499 (0.208-1.196)	0.119	0.349 (0.108-1.132)	0.080
Other combination of antihypertensives						
No	36 (27.7)	94 (72.3)	Reference			
Yes	6 (40)	9 (60)	1.741 (0.578-5.241)	0.324		
Type of therapy						
Mono-therapy	24 (30.4)	55 (69.6)	Reference			
Multi-therapy	18 (27.3)	48 (72.7)	0.859 (0.417-1.772)	0.681		
Statins						
No	12 (30)	28 (70)	Reference			
Yes	30 (28.6)	75 (71.4)	0.933 (0.420-2.073)	0.865		
Phosphate binders						
No	5 (27.8)	13 (72.2)	Reference			
Yes	37 (29.1)	90 (70.9)	1.069 (0.356-3.212)	0.906		

Analysis: Univariate and Multivariate binary logistic regression analysis. All variables with *p*-value < 0.25 will be included in the multivariate analysis. *OR* Odds ratio, *CI* confidence interval, *BMI* Body mass index, *NGO* Non-governmental organization, ^a^Other comorbidities: Blood clots, depression, asthma, osteoarthritis, pregnancy losses/birth defects and osteoporosis. *ACE-I* Angiotensin converting enzyme inhibitors, *ARB* Angiotensin receptor blocker, *CCB* Calcium channel blocker

In the multivariate logistic regression analysis, the only variable which had statistically significant association with pre-dialysis controlled hypertension was prescription of CCBs (OR = 8.988, *p*-value = < 0.001). Those patients who were receiving CCBs had significantly high rate of hypertension control than those who were not receiving it **(**Table 6).

## Discussion

Although the use of ACE inhibitors and ARBs are associated with reduction of BP in HD patients [8] limited literature is available on the evaluation of factors associated with pre-dialysis controlled hypertension among euvolemic hemodialysis patients. This is seen even though the prevalence of uncontrolled hypertension in HD patients as defined based on the recommendations by KDOQI of achieving a pre-HD systolic BP < 140 mmHg and a post-HD systolic BP < 130 mmHg, [5] is reported to be high (80–90%) [27].

The probability of combining two or more medications to achieve good targeted BP can be reduced in certain ethnic groups who are relatively more responsive to certain classes of antihypertensive drugs used for lowering BP. The fixed-dose combination therapy of certain drugs such as a CCB and ACE inhibitors are known to confer some beneficial complementary physiologic action, lower side-effect profiles, improve tolerability, compliance, and salutary effect on target organs at a relatively lower cost. To date, different types of fixed-dose combination therapies for lowering BP are available and are commonly employed for clinical use [28].

In our study, the observed positive association between prescription of CCB and predialysis controlled hypertension is similar to the findings of a randomized controlled trial on nitrendipine [10]. Similarly, the findings of another retrospective study in HD patients suggest that the use of CCBs are associated with a lower risk of mortality [29] indicating the benefits of administering CCB in HD patients. In contrast, London et al in a small clinical trial reported that a CCB named nitrendipine failed to reduce left ventricular hypertrophy as compared to the use of an ACE inhibitor (perindopril) despite having effectively lowered BP to similar levels [30]. Nevertheless, since CCBs are not removed by HD, no additional post-dialysis dosing is required. Moreover, a once daily dosing of most CCBs make them attractive for use in HD patients [31] which warrants our further investigation.

The results from an observational study by Kestenbaum et al demonstrated that CCBs contribute to a 21% lower risk of all-cause mortality and 26% cardiovascular specific mortality [11]. In addition, CCBs exhibit a variety of other potential therapeutic properties in HD patients. Vascular smooth muscle relaxation, better BP control and attenuation of heart rate as well as contractility are among the specifically important parameters for HD patients who have high incidence of hypertension and left ventricular hypertrophy [32, 33] are all purported mechanisms of action of CCBs which are useful.

A multicenter prospective study [34] conducted in Japan found that the use of benidipine, a dihydropyridine derivative calcium antagonist, alone or when added to ACE inhibitors reduced BP less than 150/90 mmHg in almost 100% patients within a month which is similar to the findings in our study. It has been reported that treatment of a group of patients with calcium antagonists do not affect urinary protein excretion, although proteinuria is significantly reduced in patients treated with ACE inhibitor. However, it was noted that although CCBs do not affect proteinuria in the treated patients, they could slow down the progression of renal insufficiency while decreasing the BP significantly [32]. Similarly, Zucchelli et al. [35] in their prospective, randomized controlled trial showed the influence of captopril (an ACE inhibitor) and nifedipine (a CCB) on BP, renal insufficiency progression and proteinuria for three consecutive years found that both treatments exhibited similar effects on the progression rate of renal failure with similar reduction in BP seen with no significant reduction in proteinuria. In addition to these results, the Systolic Hypertension in Europe (Syst-Eur) Trial (1999) demonstrated that dihydropyridine-based antihypertensive treatment is particularly beneficial in older diabetic patients with isolated systolic hypertension [36]. Taken together, the results from the current study indicates the good potential of CCBs.

In agreement to our findings, another multicenter trial [37] also indicated a rapid reduction in BP when patients were treated with CCBs. These results further support our point of view that CCBs should be incorporated into the therapy of elderly hypertensive patients with chronic renal insufficiency with careful monitoring of BP. Similarly, CCBs have been found to be effective in cases of renal failure where patients tend to exhibit significant resistance to antihypertensive medications [38]. Moreover, studies in several animal models of progressive renal failure have shown that in addition to their antihypertensive effects, CCBs have other established advantages where like other vasodilating agents, they neither cause sodium and water retention nor hyperkalemia as usually seen with ACE inhibitors administrations [39]. However, at the moment it may not be evidently claimed that the results obtained from the referred animal models could be extrapolated to humans [40].

In addition to the above, CCBs are safe and have effective roles in treating or mitigating various complications pertaining to cardiovascular disorders and renal diseases in diabetic patients. For instance, the findings of a placebo-controlled double-blind trial revealed that antihypertensive treatment employing a dihydropyridine CCB indicated some beneficial effects in older diabetic patients as compared to the non-diabetic patients which reject the hypothesis that the use of long- acting CCBs is harmful in older diabetic patients [35].

There was similar reported cardiovascular benefit in patients who receive nitrendipine alone as opposed to the use of either enalapril or hydrochlorothiazide (or both nitrendipine and either enalapril or hydrochlorothiazide) [41]. It has been reported in many outcome trials that the relative benefit of antihypertensive therapy has been similar, but there is a wide difference in the absolute benefit according to the number of outcomes observed in the control group [42]. In a randomized trial [43], it has been reported that patients receiving fosinopril experienced a significantly lower number of acute myocardial infarction or stroke or angina pectoris (14 of 189 patients, vs. 27 of 191 treated with amlodipine). However, this was an open randomized controlled trial and the adverse effects were recorded by asking patients whether they had been hospitalized or had any other discomfort.

In keeping with our findings, the Hypertension Optimal Treatment Trial [15] revealed that BP control can be achieved (target diastolic BP, 80 mmHg rather than 90 mmHg) with the use of felodipine as the first-line agent, and resulted in lower rates of all cardiovascular events in 1501 study participants with diabetes (relative risk, 0.49; 95% confidence interval, 0.29 to 0.81; *p* = 0.005) but not in the overall study participants of 18,790 patients (relative risk, 0.93; 95% confidence interval, 0.78 to 1.12; *p* = 0.50). Similarly, the effects of 5 to 20 mg/day of manidipine, a dihydropyridine-type CCB on seventy- one renal impairment hypertensive patients on their BPs and renal functions were investigated for more than 48 weeks [37]. In our study BP was well controlled in 25 (58.1%) patients out of 43 patients thus highlighting the potential benefits of CCBs in euvolemic hypertensive HD patients. Therefore, careful selection of antihypertensive drugs in these special group of patients are recommended.

## Conclusion

Our study revealed a positive association between pre- dialysis controlled hypertension among euvolemic hypertensive patients and prescription of CCBs. However, the results of the current study should be interpreted with the major limitations of limited sample size and lack of information about patients’ adherence with antihypertensive medications and life style interventions. A large multi-center prospective study is recommended to confirm the present findings.

### Study limitations

The findings of the present study need to be interpreted with caution since it is limited to only 6 months follow up. Nevertheless, a multicenter study with a large sample size and longer follow-up time is needed to confirm the findings of the current study. Furthermore, some other factors that affect blood pressure control such as salt intake, exercise, etc. were not assessed in this study. As the study was carried out in Kelantan, Malaysia, where Malays are the predominant inhabitants. Malaysia is multiethnic country with the three predominate ethnicities i.e. Malays, Chinese and Indians. The results of this study therefore cannot be extended to the whole population of the country.
